# District of Columbia

**Published:** 1896-07

**Authors:** 


*District of Columbia.*—The bill destined to secure for the District of Columbia a Board of Veterinary Examiners, and requiring registration upon all practitioners in the District, failed to pass at the recent session of Congress.

